# Caffeine addiction: optimising neonatal caffeine use

**DOI:** 10.1038/s41390-026-04799-7

**Published:** 2026-02-04

**Authors:** Eleanor J. Molloy, Joanne O. Davidson, Nicoleta Barbu, Alistair J. Gunn

**Affiliations:** 1 Discipline of Paediatrics, Trinity College, University of Dublin, Dublin, Ireland; 2 Trinity Translational Medicine Institute, Dublin, Ireland; 3 Trinity College Institute of Neuroscience, Trinity College Dublin, Dublin, Ireland; 4 Trinity Research in Childhood Centre (TRiCC), Trinity College Dublin, Dublin, Ireland; 5 Paediatrics Department, Coombe Hospital, Dublin, Ireland; 6 Neonatology Department, Children’s Health Ireland, Dublin, Ireland; 7 Neurodisability Department, Children’s Health Ireland, Dublin, Ireland; 8 Department of Physiology, Auckland University, Auckland, New Zealand

Caffeine is the most widely used stimulant in humans and has become ubiquitous in neonatal intensive care following the key Caffeine for Apnoea of Prematurity (CAP) trial.^1^ In this study, Schmidt et al. randomized 2006 very low birth weight (VLBW) infants to receive either caffeine or placebo until treatment for apnoea was no longer required.^1^ There were significant improvements in motor outcomes at 2 years and fine motor development at 5 years, including enhanced visuomotor, visuoperceptual, and visuospatial functions with reduced developmental coordination disorder^1–3^ and benefits persisting through 11 years of age.^4^ In 2010, the Cochrane review by Henderson-Smart and De Paoli found significant decreases in apnoeic episodes with caffeine administration, reducing the need for mechanical ventilation and improving outcomes.^5^ Similarly, metanalysis by Alhersh et al suggested that although mortality was not reduced, there was a significant decrease in the need for mechanical ventilation.^6^ A recent comprehensive meta-analysis by Oliphant et al. confirmed caffeine’s effectiveness in reducing apnoea and preventing neurodevelopmental impairment.^7^

Caffeine is a methylxanthine and exerts pleiotropic effects, including immunomodulatory actions and antagonism of adenosine receptors, which play a key role in suppressing respiratory drive. The increased oxygen consumption associated with methylxanthines, including caffeine, may have significant impacts on multiorgan growth and function in preterm infants.^8–12^

In their recent study published in this journal, Ostrem et al.^13^ investigated the relationship between cumulative caffeine exposure and neurodevelopmental outcomes. Among the primary cohort of 67 preterm infants, higher caffeine doses were associated with improved motor, language, and cognitive performance on the Bayley-III assessment. These findings remained significant after adjusting for gestational age and respiratory disease. However, these associations were not observed in the larger cohort of 106 infants, suggesting that while higher maintenance dosing may confer neurodevelopmental benefits, the evidence remains inconclusive, necessitating further investigation to establish optimal dosing strategies.

## Multiorgan considerations

Caffeine impairs cerebral and intestinal blood flow velocity in preterm infants, raising concerns about potential ischemic organ injury.^14^ Caffeine influences renal perfusion and diuresis, which may impact fluid and electrolyte balance in neonates. These effects warrant careful monitoring, especially in the context of prolonged or high-dose therapy. As an immunomodulator, caffeine may alter inflammatory responses, although the clinical significance of this in preterm infants remains under investigation. The expanding use of caffeine beyond apnoea treatment—sometimes referred to as “therapeutic creep”—highlights the need to reassess dosing and timing to maximize benefits while minimizing risks.^15,16^

## The future

Clinical trials of caffeine in small animal models suggest that caffeine is neuroprotective in neonatal hypoxic ischemic brain injury and therefore may be extended to clinical trials in term infants with encephalopathy. However, it is critical to appreciate that adenosine levels increase dramatically during profound hypoxia-ischemia, improve local cerebral blood flow, suppress neuronal activity, and so help to delay the onset of anoxic depolarization and reduce neuronal loss.^17^ This active endogenous neuroprotection likely contributes to the remarkable tolerance of the fetus to hypoxia-ischemia. Similarly, seizures are associated with local tissue hypoxia, with a dramatic increase in adenosine release, which ultimately helps to terminate the seizure event^18^ and, conversely, other methylxanthines such as theophylline exacerbate seizures.^19^ Given this background, concern has been raised that the apparent benefit of caffeine during induced inhalational hypoxia in small animals may be mediated by confounding support of respiration, and therefore, the use of any adenosine receptor antagonist such as caffeine may be detrimental. This strongly suggests that further studies are required before translation to clinical trials. Finally, although high doses of caffeine control apnoea more effectively, there are concerns about off target effects such as inhibition of cholinergic and GABA receptors as well as effects on phosphodiesterase, adrenergic, and dopaminergic systems.^20^ Further exploration of pharmacogenomics in preterm neonates may help to define the clinical impact.

## Conclusion

Caffeine remains a vital therapy in neonatal care, with strong evidence supporting its neurodevelopmental benefits for preterm infants. However, its multiorgan effects, particularly on cerebral blood flow and metabolisms, renal function, and immune modulation, underscore the importance of individualized dosing and ongoing evaluation. We strongly advocate that we should resist therapeutic creep to other indications without compelling evidence base. Future studies should aim to refine caffeine therapy to optimize outcomes while safeguarding against potential adverse effects.
